# Early-Life Programming and Reprogramming of Adult Kidney Disease and Hypertension: The Interplay between Maternal Nutrition and Oxidative Stress

**DOI:** 10.3390/ijms21103572

**Published:** 2020-05-18

**Authors:** Chien-Ning Hsu, You-Lin Tain

**Affiliations:** 1 Department of Pharmacy, Kaohsiung Chang Gung Memorial Hospital, Kaohsiung 833, Taiwan; chien_ning_hsu@hotmail.com; 2 School of Pharmacy, Kaohsiung Medical University, Kaohsiung 807, Taiwan; 3 Department of Pediatrics, Kaohsiung Chang Gung Memorial Hospital and Chang Gung University College of Medicine, Kaohsiung 833, Taiwan; 4 Institute for Translational Research in Biomedicine, Kaohsiung Chang Gung Memorial Hospital and Chang Gung University College of Medicine, Kaohsiung 833, Taiwan

**Keywords:** antioxidant, developmental origins of health and disease (DOHaD), hydrogen sulfide, hypertension, kidney disease, nitric oxide, nutrition, offspring, oxidative stress, pregnancy, reprogramming

## Abstract

Kidney disease and hypertension both have attained the status of a global pandemic. Altered renal programming resulting in kidney disease and hypertension can begin in utero. Maternal suboptimal nutrition and oxidative stress have important implications in renal programming, while specific antioxidant nutrient supplementations may serve as reprogramming strategies to prevent kidney disease and hypertension of developmental origins. This review aims to summarize current knowledge on the interplay of maternal nutrition and oxidative stress in response to early-life insults and its impact on developmental programming of kidney disease and hypertension, covering two aspects. Firstly, we present the evidence from animal models supporting the implication of oxidative stress on adult kidney disease and hypertension programmed by suboptimal maternal nutrition. In the second part, we document data on specific antioxidant nutrients as reprogramming strategies to protect adult offspring against kidney disease and hypertension from developmental origins. Research into the prevention of kidney disease and hypertension that begin early in life will have profound implications for future health.

## 1. Introduction

The epidemic of kidney disease and hypertension poses a major public health challenge around the world [1,2]. Hypertension can be both a cause and a result of kidney disease. A growing body of evidence indicates that the origins of both kidney disease and hypertension can initiate in early life [3,4]. This concept has been denoted as the “developmental origins of health and disease (DOHaD)” after observing the enduring effects of the early-life suboptimal environment on adverse health outcomes in adulthood [5].

**Figure 1:**
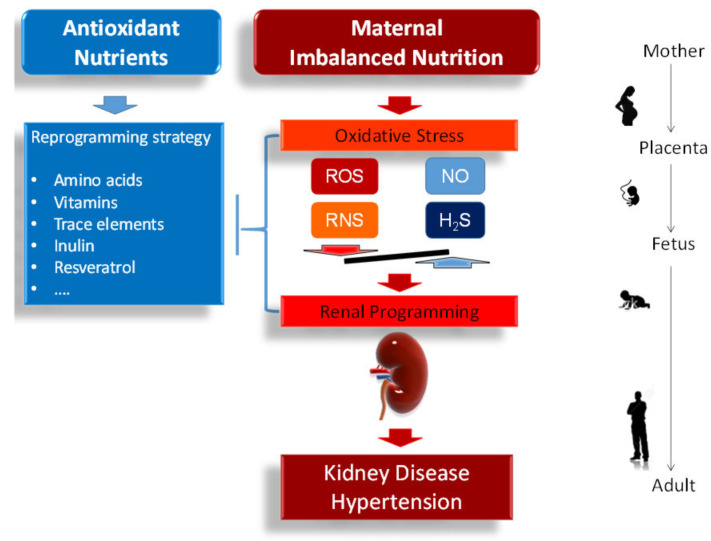
Schematic illustration of the association between maternal nutrition, oxidative stress, renal programming, and increased vulnerability to kidney disease and hypertension in adult offspring. Red arrow indicates imbalanced nutrients in pregnancy and lactation causes oxidative stress and renal programming, consequently leading to kidney disease and hypertension in adult offspring. There are several signaling systems for maintaining the redox balance, including reactive oxygen species (ROS), reactive nitrogen species (RNS), nitric oxide (NO), and hydrogen sulfide (H_2_S). Conversely, certain antioxidant nutrients can serve as reprogramming strategies to reverse the programmed processes and prevent the developmental programming of kidney disease and hypertension, which is indicated by blue arrow and lines.

During kidney development, the renal structure and function can be permanently altered in response to a variety of adverse intrauterine environmental factors by so-called renal programming [6,7]. One of the most characteristic insults is maternal malnutrition [8,9]. The developing kidney can be programmed by a poor nutritional environment in pregnancy, leading to kidney disease and hypertension in later life [3,8,10]. Likewise, oxidative stress, an imbalance between reactive oxygen species and antioxidant defenses, plays a pathophysiological role in fetal programming [11,12,13]. Emerging evidence demonstrates that early-life oxidative stress can increase the risk of developing kidney disease and hypertension in later life [3,7]. Conversely, nutritional programming can also be beneficial [10]. Certain antioxidant nutrients during the period of developmental plasticity can reverse the programming processes to prevent various adult diseases, also known as reprogramming [14,15]. This review highlights the interplay between nutrition and oxidative stress in pregnancy, causing renal programming, links developmental programming of kidney disease and hypertension. Particular interest is given to the perinatal use of antioxidant nutrients as reprogramming strategies to reverse renal programming and prevent kidney disease and hypertension of developmental origins. The interplay among maternal nutrition, oxidative stress, renal programming, and the risks for developing kidney disease and hypertension in adult offspring is illustrated in Figure 1.

Relevant peer-reviewed journal articles published in English were identified in the MEDLINE/PubMed databases. Different combinations of the following keywords were used: “kidney disease”, “hypertension”, “blood pressure”, “developmental programming”, “DOHaD”, “offspring”, “progeny”, “pregnancy”, “mother”, “nephrogenesis”, “nutrition”, “nutrients”, “oxidative stress”, “antioxidant”, “nitric oxide”, “hydrogen sulfide” and “reprogramming”. The last search was conducted on 1 April 2020. Bibliographic references from eligible articles were reviewed for the selection of any additional studies.

## 2. Maternal Nutrition and Renal Programming

### 2.1. Kidney Development

The functional unit of the kidney is the nephron. The human kidney has around 1,000,000 nephrons per kidney, with a 10-fold difference among individuals [16]. Nephrogenesis in humans occurs between the 9th and 36th week of gestation [17]. Metanephric kidney development initiates when a ureteric bud outgrowth from the nephric duct invades a group of mesenchymal cells in the nephric cord. This process of ductal elaboration is known as branching morphogenesis [18], which leads to the formation of the nephrons and urinary collecting system. Nephron progenitors epithelialize to form the renal vesicle, which further develops into the S-shaped body before fully developing into a nephron. Thus, impaired branching morphogenesis may lead to low nephron endowment and a wide spectrum of clinical phenotypes, namely congenital anomalies of the kidney and urinary tract (CAKUT). By the end of gestation, nephron development is complete between 32 and 36 weeks [16]. Thus, full-term infants normally get a complete endowment of nephrons. However, nephron number in preterm infants largely depends on gestational age, intrauterine environment, and perinatal care. Low birth weight due to premature birth or intrauterine growth restriction is associated with low nephron number [16,17,19]. Several epidemiologic studies support that prematurity and low birth weight are risk factors for kidney disease and hypertension in later life [20,21,22]. Nevertheless, little is known about the impact of imbalanced nutrition during pregnancy on nephron endowment and renal programming in human studies.

### 2.2. Animal Models for Maternal Nutritional Insults-Induced Renal Programming

Maternal nutrition is the major intrauterine factor that regulates fetal growth and organ development. The developing kidney is particularly susceptible to the effects of suboptimal maternal nutrition [6,7]. Insufficient calorie intake or deficiency of a particular nutrient in pregnancy has been linked to the developmental programming of many adult diseases [9]. On the other hand, overnutrition is another type of malnutrition in which the intake of nutrients or a specific nutrient is oversupplied, especially in unbalanced proportions. Table 1 summarizes animal studies demonstrating the association between early-life nutritional insults and subsequent hypertension and kidney disease in adult offspring [23,24,25,26,27,28,29,30,31,32,33,34,35,36,37,38,39,40,41,42,43,44,45,46,47,48,49,50,51,52,53,54,55,56,57,58,59,60,61,62]. The current review is solely confined to nutritional insults starting in pregnancy and/or lactation period in rodent models. Rodents, usually rats and mice, are currently the most commonly used animals for DOHaD research. Unlike in humans, nephrogenesis in rodents continues up to postnatal week 2. Accordingly, suboptimal environmental factors during pregnancy and early lactation period may disturb kidney development, leading to renal programming and kidney disease in adulthood. Rats grow rapidly during their childhood and reach sexual maturity at about six weeks of age. In adulthood, one rat month equals to three human years [63]. Hence, Table 1 lists the timing of developing kidney disease and hypertension evaluated at different ages, which allows calculations to refer to humans of a specific age group.

### 2.3. Nephron Number and Maternal Suboptimal Nutrition

As shown in Table 1, different types of suboptimal nutrition in pregnancy and/or lactation have been shown to be capable of inducing several phenotypes of renal programming in adult offspring, including elevation of BP, glomerular hypertrophy, glomerular and tubulointerstitial injury, changes of glomerular filtration rate (GFR), proteinuria, and altered renal transcriptome. Additionally, certain early-life nutritional insults can cause low nephron endowment, as in the case of maternal caloric restriction [25,26], maternal protein restriction [34,36], iron deficiency [54], and multideficient diet [60]. However, nephron endowment can also be unaltered [37,50], or even increased in response to maternal imbalanced nutrition-induced renal programming [62]. Thus, low nephron endowment, per se, does not appear to be an indispensable mechanism underlying programmed processes of kidney disease. A reduction of nephron number, in the presence of compensatory hypertrophy, would be expected to counteract a decreased GFR. Interestingly, variations of GFR observed in different models of renal programming can be unaltered [25,30,36,37], reduced [46,61], or even increased [34,60,62]. These data indicate that there is a different level of compensation in the setting of a low nephron endowment in response to various nutritional insults.

### 2.4. Renal Transcriptome and Maternal Suboptimal Nutrition

Currently, only a few genome-wide studies have been conducted to determine the alterations in the renal transcriptome in developmental models of hypertension, with a focus on early-life nutritional insults [31,46,48,56]. Using next-generation RNA sequencing (NGS) analysis, a total of 2706 differential expressed genes (DEGs) (1214 up- and 1492 down-regulated genes) were identified in 1-day-old male offspring kidney in the maternal high-fructose diet model [31]. Among them, *Slit2, Spry3, Pod1, Col4a1, Col4a2, Col4a4, Wnt9b, Lif, Fgf2, Fgf20, Notch2, Jnk3,* and *Cdh6* were involved in nephrogenesis. NGS also identified genes in arachidonic acid metabolism (*Cyp2c23, Hpgds, Ptgds,* and *Ptges*) that may be potential genes/pathways contributing to renal programming and hypertension. In another programming model, maternal high-fat diet-induced significant changes in renal transcriptome with female offspring being more sensitive [48]. Additionally, maternal high-salt diet modified 272 DEGs in a 2-week-old offspring kidney, right after the completion of nephrogenesis [48]. Compared with three other programmed hypertensive models, there were 16 shared genes that are related to the regulation of BP included *Adrb3, Alb, Apoe, Calca, Kng1, Adm2, Guca2b, Hba2, Hba-a2*, and *Ppara* [48]. Moreover, a previous report showed maternal methyl-deficient diet and high methyl-donor diet altered respectively 938 and 806 renal transcripts in adult offspring [56]. Although there was a total of 201 DEGs were shared by two different nutritional insults, none of them have shown a direct relationship with hypertension [56]. Although renal transcriptome seems to be crucial in renal programming, the roles of DEGs identified by different models deserve further clarification.

### 2.5. Maternal Macronutrients Intake and Renal Programming

The main phenotype of renal programming is hypertension. Dietary nutrients can be divided into macronutrients, micronutrients, and non-essential nutrients. Carbohydrates, proteins, and fats are macronutrients, which has a number of calories to provide our body with energy. Caloric restriction ranging from 30%–70% to pregnant dams resulted in elevated BP, glomerular hypertrophy, and tubulointerstitial injury in the adult offspring [23,24,25,26,27,28]. Pups exposed to a more severe degree [27] or longer exposure times [25] of caloric restriction were likely to develop hypertension earlier. Moderate (30%) caloric restriction during pregnancy caused hypertension in adult offspring at 54 weeks of age [23], which is equivalent to human middle adulthood. While severe (50%) caloric restriction during pregnancy and lactation periods raised offspring BP at as early as 12 weeks of age, which equals roughly to young adulthood in humans.

Sugar consumption, particularly fructose, has paralleled an increase in hypertension and obesity during the past several decades [64]. Consumption of high-fructose with various amounts and duration by rodent mothers showed the rise in BP in adult offspring ranging from 12 to 52 weeks of age [30,31,32]. These observations suggest that offspring exposed to higher fructose concentration tend to develop hypertension at an earlier age. Unlike carbohydrates, previous reports studying the programming effects of another macronutrient protein are mainly focused on deficient instead of excessive intake. Rat models of low protein feeding have been extensively used to study the mechanisms of nutritional programming [65]. In rodents, offspring exposed to protein restriction ranging from 6%–9% during pregnancy and/or lactation period develop hypertension [33,34,35,36,37,38,39,40,41]. It appears that a large scale of early-life protein restriction shows a higher propensity to induce hypertension earlier, even in childhood [38]. Furthermore, in utero high-fat diet is a commonly used animal model to induce the developmental programming of obesity-related disorders, like hypertension [66]. Although most studies demonstrating adult offspring exposed to a maternal high-fat consumption had a rise in BP [42,43,44,45], some studies did not support this notion [43,46]. Thus, maternal high-fat diet may or may not induce programmed hypertension in adult offspring is likely related to sex, strain, age, and the variations of fatty acid compositions.

Moreover, sodium, potassium, calcium, magnesium, and other ions are also listed with macronutrients as they are needed in large quantities. Both low- and high-salt diet exposure during pregnancy and lactation have been shown to induce hypertension in male adult offspring [47,48]. Maternal calcium-deficient diet increased BP in adult offspring [49], while magnesium-deficient diet did not [50]. A moderate level of dietary magnesium deficiency in pregnancy did not program for a nephron deficit, but increased urine flow and reduced magnesium excretion [50].

### 2.6. Renal Programming Related to Maternal Micronutrients Intake and Imbalanced Nutrients

Evidence also indicates that deficiencies in certain micronutrients, namely zinc [51], iron [52,53,54], and vitamin D [55], lead to elevated BP in male adult offspring. Of note, maternal iron restriction caused the rise in female offspring BP across a wide age range from 10 weeks to 18 months of age [52,54], at which approximately women experience menarche to menopause in humans.

Instead of focusing on one particular nutrient, current evidence suggests that imbalanced nutrition, which considers how mixtures of nutrients also link to developmental programming of kidney disease and hypertension. The methionine, choline, folic acid, vitamins B2, B6, and B12 have an essential role as donors or coenzymes in one-carbon metabolism, serving as methyl-donor nutrients. Methyl-donor food has been recommended for pregnant women to reduce adverse birth outcomes [67]. Our previous report showed that pregnant rats received a high-methyl-donor diet caused hypertension in male adult offspring [56]. Interestingly, we found that adult offspring exposed to maternal methyl-deficient diet also developed hypertension [56]. Thus, additional studies should be taken to assess the programming effects in regards to the maternal methyl-donor diet.

Also, some studies used fructose, fat, or salt as a part of the maternal diet along with others [57,58,59]. It is well known that the Western diet is characterized by the intake of high-sugar drinks, high-fat products, and excess salt. Indeed, animal studies examining the combined effects of key components of the Western diet have shown their synergistic effects on the programmed hypertension in adult offspring [59,68,69]. Thus, the interplay between carbohydrate, fat, and salt on the programming of hypertension and kidney disease deserves further clarification. Moreover, adult offspring exposed to two completely different nutritional insults, multi-deficient diet [60,61] vs. early postnatal hypernutrition [62], both induce similar phenotypes—hypertension and glomerular hypertrophy. These data suggest there might be some common mechanisms contributing to the pathogenesis of renal programming.

## 3. Oxidative Stress, Maternal Suboptimal Nutrition, and Renal Programming

### 3.1. Oxidative Stress is a Common Mechanisms of Renal Programming

Animal models have provided insight on several common mechanisms underlying renal programming, for example, oxidative stress, alterations of the renin-angiotensin system (RAS), nutrient-sensing signals, and epigenetic regulation [3,4,6,7,10,12]. Among them, oxidative stress acts as a hub and connects to other important mechanisms involved in programmed hypertension and kidney disease [3,7,9,10,12].

**Figure 2:**
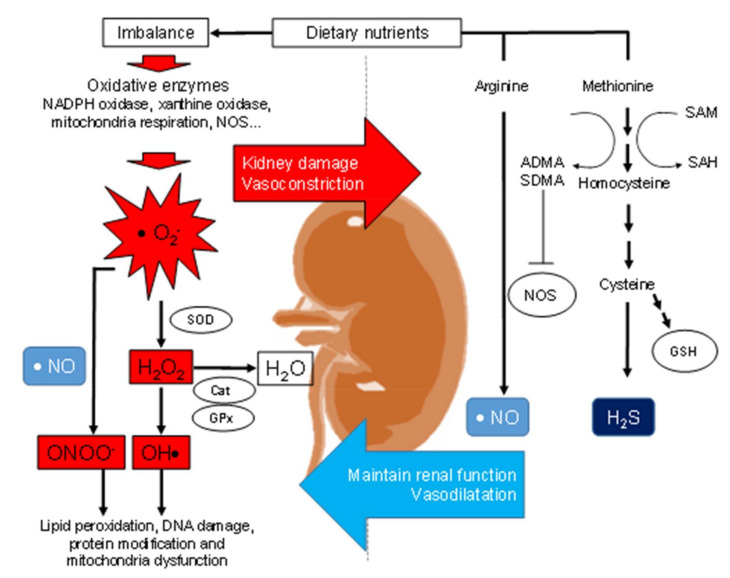
Schematic representation of the interrelationships between reactive oxygen species (ROS, antioxidant defense system, nitric oxide (NO), and hydrogen sulfide (H_2_S) signaling system in the kidney to control blood pressure and renal function. Imbalanced nutrition can activate reactive oxygen species (ROS) and reactive nitrogen species (RNS), leading to kidney damage and vasoconstriction. Several oxidative enzymes can produce superoxide anion (O2^−^), such as NADPH oxidase, xanthine oxidase, and mitochondrial respiration. The superoxide anion can interact with nitric oxide (NO) to form peroxynitrite (ONOO^−^). Hydrogen peroxide (H_2_O_2_) and hydroxyl anion (OH^−^) are reactive byproducts of superoxide. These ROS can be produced via superoxide dismutase (SOD), catalase, or glutathione peroxidase (GPx). On the other hand, dietary nutrients provide L-arginine and L-methionine for the synthesis of NO and H_2_S, respectively. Both NO and H_2_S are vasodilators and play a key role in maintaining renal physiology. While L-arginine can also be posttranslational methylation to form asymmetric or symmetric dimethylarginine (ADMA or SDMA), both are endogenous inhibitors of nitric oxide synthase (NOS). There is a relationship between the L-arginine-NO and the H_2_S-synthesizing pathway. The protein arginine methyltransferases (PRMTs) methylate L-arginine to generate ADMA/SDMA and concurrently demethylate methionine to form homocysteine. S-adenosylmethionine (SAM) is used as a substrate for methyl transfers that yield S-adenosylhomocysteine (SAH). Homocysteine can further be broken down to generate L-cysteine and glutathione.

The interrelationships between maternal nutrition, oxidative stress, and renal programming are illustrated in Figure 2. Oxidative stress reflects an imbalance between reactive oxygen species (ROS) and antioxidant defenses. Nitric oxide (NO) is a potent vasodilator, as well as a free radical, also plays a key role in oxidative stress [70]. NO deficiency and increased oxidative stress in the kidney is involved in the pathogenesis of hypertension [70]. Like NO, hydrogen sulfide (H_2_S) also acts as a vasodilator and antioxidant, playing a key role in oxidative stress and renal programming [71,72]. Early-life oxidative stress is associated with an increase in risk for hypertension in adulthood [11,12,13]. Several nutritional insults in pregnancy and lactation have been reported to induce renal programming attributed to oxidative stress, namely caloric restriction [25], maternal high-fructose diet [30], maternal protein restriction [39], high-fat diet [44,45,46], maternal zinc restriction [51] and maternal methyl-donor diet [56].

### 3.2. Renal ROS, Renal Programming, and Programmed Hypertension

ROS, such as superoxide anion (O_2_^−^), hydrogen peroxide (H_2_O_2_), and hydroxyl anion (OH^−^), are reactive byproducts of oxidases enzymes, such as nicotinamide adenine dinucleotide phosphate (NADPH) oxidase, xanthine oxidase, and mitochondrial respiration [73]. In certain conditions, superoxide can also be formed by nitric oxide synthases (NOS); for example, depletion of the NOS substrate L-arginine, depletion of the cofactor tetrahydrobiopterin (BH_4_), or during inhibition of NOS by asymmetric dimethylarginine (ADMA) [74]. The superoxide can react rapidly with NO to form peroxynitrite (ONOO^−^). Peroxynitrite is the most reactive and potentially injurious reactive nitrogen species (RNS). Conversely, O_2_^−^ can be removed by superoxide dismutase (SOD) to form H_2_O_2_. Subsequently, H_2_O_2_ can be metabolized by several antioxidant enzymes like catalase, peroxiredoxins, and glutathione peroxidase (GPx) [73]. Increased renal ROS production contributes to the development of hypertension by inducing renal vasoconstriction, activating the RAS, increasing renal afferent nerve activity, or reducing vasodilator production [74].

Furthermore, oxidative stress causes kidney damage by promoting lipid peroxidation, DNA damage, protein modification, and mitochondrial dysfunction [75]. These processes have been implicated in the pathogenesis of programmed kidney disease and hypertension. Maternal caloric restriction caused by an elevation of BP in adult offspring is associated with increased superoxide production [24]. Another report showed that hypertension programmed by maternal low-protein intake was related to increased renal 8-isoprostaglandin F2α level (a biomarker of lipid peroxidation) and decreased antioxidant glutathione level [39]. In a maternal high-fat diet model, adult offspring developed hypertension combined with increased malondialdehyde levels and decreased activities of antioxidative enzyme SOD, catalase, and GPx [42]. Additionally, increased 8-hydroxydeoxyguanosine (8-OHdG), an oxidative stress-induced DNA damage marker, expression in offspring kidneys has been reported in several models of programmed hypertension. These models, such as maternal high-fat consumption [46], maternal methyl-deficient diet [56], maternal methyl-donor diet [56], and maternal high-fructose intake [68], are all relevant to maternal nutritional insults. Moreover, increased renal NADPH oxidase subunit expression and lipid peroxidation have been reported in adult offspring born to dams treated with multideficient diet in pregnancy, which displayed hypertension and renal dysfunction [61]. Together these observations indicate that renal ROS plays a key role not only in renal programming but also in hypertension of developmental origins.

### 3.3. Arginine-ADMA-NO Pathway in Renal Programming

Emerging evidence supports that early-life NO–ROS imbalance is capable of programming kidney, leading to hypertension and kidney disease in adulthood [76,77]. As we reviewed elsewhere [78], impaired arginine–ADMA–NO pathway is closely interrelated to oxidative stress in determining the programming process. NO is generated from the conversion of L-arginine to L-citrulline by NOS. Protein-incorporated arginine can be posttranslational methylation to form ADMA or symmetric dimethylarginine (SDMA) by a family of protein arginine methyltransferases (PRMTs) [74]. Both ADMA and SDMA are known to inhibit NO production. ADMA can be metabolized by dimethylarginine dimethylaminohydrolase-1 (DDAH-1) and -2 (DDAH-2).

Oxidative stress can reduce NO production by oxidizing cofactor BH4 to uncouple NOS, inhibiting DDAH activity from increasing ADMA or scavenging NO by superoxide to form peroxynitrite [78]. Therefore, inactivation of NO by oxidative stress in the kidney may, at least in part, contribute to the developmental programming of hypertension and kidney disease. Indeed, increased plasma ADMA levels and decreased NO bioavailability have been found in offspring hypertension programmed by maternal caloric restriction [25], high-fructose consumption [30], and high-fat intake [46].

NO depletion in pregnancy-induced by N^G^-nitro-L-arginine-methyl ester (L-NAME, an inhibitor of NOS) led to renal programming, along with increased oxidative stress in adult offspring [79]. During nephrogenesis, maternal NO deficiency modified more than 2000 renal transcripts in a 1-day-old offspring kidney. Among them, several genes belonging to the RAS (e.g., *Agt, Ace2, Agtr1a*, and *Mas1*) and arachidonic acid metabolism pathway (e.g., *Cyp4a2, Hpgds, Ptgds, Ptgs1, Ptgs2*, and *Ephx2*) are involved in the pathogenesis of programmed hypertension [79].

Of note is that ADMA can impair nephrogenesis [80]. Metanephroi grown in 2 or 10 µM ADMA were significantly smaller and contained lower nephron numbers in a dose-dependent manner [80]. We used to identify 1221 differential expressed genes in cultured metanephroi treated with 10 µM ADMA [48] by NGS analysis. Among them, *Avpr1a, Ephx2, Hba2, Hba-a2,* and *Npy1r* have been reported in association with programmed hypertension in other models [30,81]. Accordingly, these observations support the notion that impaired L-arginine-ADMA-NO pathway in the kidney contributes to the developmental programming of hypertension and kidney disease.

### 3.4. H_2_S Pathway in Renal Programming

H_2_S has multifaceted biofunctions comprising angiogenesis, vasodilatation, anti-inflammation, antioxidant, mitochondria bioenergetics, metabolic modulation, renal excretory function, and antiapoptosis [82,83]. In the kidney, enzymatic synthesis of H_2_S from L-cysteine occurs through three enzymes, cystathionine β-synthase (CBS), cystathionine γ-lyase (CSE), and 3-mercaptopyruvate sulphurtransferase (3MST) [82]. CBS catalyzes homocysteine and serine to generate cystathionine. Later CSE breaks down cystathionine into L-cysteine, α-ketobutyrate, and ammonia. Also, CSE catalyzes L-cysteine to produce pyruvate, H_2_S, and ammonia. The beneficial effects of H_2_S signaling have been demonstrated in many disorders, including hypertension and kidney disease [72,82,83]. L-cysteine or N-acetylcysteine (NAC, a stable cysteine analog) has been reported to prevent hypertension in experimental models by decreasing oxidative stress and modulating NO [79,84,85]. In a maternal NO deficiency model, the protective effects of NAC on adult offspring against programmed hypertension were associated with an increase in H_2_S-generating enzymes and H_2_S synthesis in the kidneys [79]. In another study, maternal NAC therapy also protected offspring against hypertension programmed by prenatal dexamethasone and postnatal high-fat diet via increases of H_2_S-generating enzymes and plasma glutathione level, and reduction of oxidative stress in offspring kidneys [85].

Of note, the L-arginine-ADMA-NO pathway is interrelated with the H_2_S synthesizing pathway. The PRMTs methylate L-arginine to generate ADMA and concurrently demethylate methionine to form homocysteine (Figure 1). Homocysteine can be further metabolized to L-cysteine and glutathione. Although the reprogramming effects of several H_2_S precursors have been studied in some developmental programming of hypertension and kidney models [72], there remains a lack of data regarding their impact in response to nutritional insults.

## 4. Antioxidant Nutrients Supplementation as Reprogramming Strategies

Antioxidant enzymes such as SOD, catalase, and GPx (including their cofactors selenium, zinc, and iron), sulfhydryl group donors (i.e., L-cysteine, L-methionine, and glutathione), and vitamins (i.e., vitamins C and E) form a network of functionally overlapping defense mechanisms against oxidative stress. These antioxidant nutrients are found in certain foods and may have beneficial effects on human health by improving oxidative stress [86]. Dietary supplements with macro- and micro-nutrients during pregnancy and lactation periods have been recommended to improve maternal and birth outcomes [87,88]. However, little is known whether supplementing with specific antioxidant nutrients in pregnancy and/or lactation can be beneficial on hypertension and kidney disease programmed by various early-life insults in humans. Here we aim to present the available data regarding antioxidant nutrients used as reprogramming strategies to prevent hypertension and kidney disease in rodent models, all of which are listed in Table 2 [24,25,27,38,45,61,79,80,85,89,90,91,92,93,94,95,96,97,98,99,100,101].

### 4.1. Amino Acids

Several amino acids have been proven to exhibit antioxidant properties and be applicable to the treatment of hypertension and kidney disease of developmental origins. First, maternal glycine supplementation protects male adult rat offspring against hypertension programmed by maternal protein restriction [38]. Second, L-citrulline supplementation has been used in pregnancy and lactation as reprogramming interventions to protect adult offspring against hypertension in several rat models, including maternal L-NAME exposure [89], streptozotocin-induced diabetes [80], and prenatal dexamethasone exposure [90]. L-citrulline is found in some foods like watermelons and is also made naturally by our body [102]. As L-citrulline is a precursor of L-arginine, it can be converted to L-arginine in the kidney for supplying NO production. Unlike L-arginine, it bypasses hepatic metabolism and it is not a substrate of arginase. Thus, oral L-citrulline supplementation has been considered as an add-on therapy to raise plasma arginine concentration and increase NO production [103]. In a maternal caloric restriction model [25], the decreases of nephron number and renal function in adult rat offspring were restored by maternal L-citrulline supplementation. Third, Table 2 shows maternal taurine supplementation protects adult offspring against hypertension programmed by maternal high-sugar diet [91] and maternal streptozotocin-induced diabetes [92]. Taurine is the most abundant sulfur-containing amino acid [104]. Although L-taurine can be synthesized by L-cysteine, it is primarily attained from dietary sources. The antihypertensive effects of L-taurine have been linked to regulation of the NO, H_2_S, the RAS, and oxidative stress [105,106]. Nevertheless, long-term reprogramming effects of maternal taurine supplementation on programmed hypertension and kidney disease still awaits further elucidation. Fourth, several studies reported NAC, a stable analog of cysteine, can reduce oxidative stress and prevent programmed hypertension in various models, as in the case of maternal L-NAME exposure [79], prenatal dexamethasone and postnatal high-fat diet [85], suramin-induced pre-eclampsia [93], and maternal nicotine exposure [94]. Last, branched-chain amino acids (BCAAs) supplementation in pregnancy was able to prevent hypertension programmed by maternal caloric restriction [27]. On the other hand, other macronutrients like carbohydrate and fat are less used as reprogramming interventions in programmed hypertension and kidney disease. Conjugated linoleic acid is a fatty acid found in meat and dairy. Only one report showed that conjugated linoleic acid supplementation during pregnancy and lactation attenuated hypertension programmed by maternal high-fat diet [95]. Although there is ample evidence leading to the conclusion that long-chain omega 3 polyunsaturated fatty acid (PUFAs, e.g., eicosapentaenoic acid (EPA) and docosahexaenoic acid (DHA)) may prevent the rise in BP and improve established hypertension [107], but their effects on programmed hypertension have not been studied yet.

### 4.2. Vitamins and Trace Elements

Vitamin C, E, folic acid, and selenium have shown BP benefits [108]. Vitamin C, E, and selenium have antioxidant properties. Folic acid is involved in DNA methylation. Gestational supplementation of combined micronutrients with vitamins C, E, selenium, and folic acid can prevent hypertension programmed by maternal caloric restriction via reducing oxidative stress [24]. Vitamin C supplementation alone in pregnancy also had BP-lowering effect on adult offspring exposed to prenatal lipopolysaccharide administration [96]. Additionally, maternal multideficient diet-induced hypertension and renal dysfunction were reported to be protected by vitamin E supplementation in lactation [61]. Also, folic acid supplementation was able to have BP benefits on adult offspring born to mothers who received a protein restriction diet [97]. Moreover, selenium supplementation during pregnancy and lactation was reported to prevent offspring against hypertension and kidney disease in a maternal methimazole exposure model [98].

### 4.3. Dietary Fiber

Dietary fiber is not absorbed by our digestive tract, whereas it is crucial in the digestion and maintenance of gut health. Maternal supplementation with dietary fiber inulin was reported to protect adult offspring against hypertension programmed by maternal high-fat diet [45] or high-fructose diet [99]. Currently, the consumption of dietary fiber has become one dietary strategy for modulating the gut microbiota. There is now growing evidence shows that gut microbiota dysbiosis in early-life might be correlated with adverse offspring outcomes, such as hypertension [109]. Given that recent studies demonstrating that microbiota-targeted therapies can be applied to a variety of diseases [110], additional studies are urgently required to explore their roles on nutritional programming-related disorders, especially the use of prebiotics in pregnancy.

### 4.4. Nutraceuticals

A nutraceutical is any substance that is a food or a part of the food and provides medical or health benefits [111]. It was concluded that certain nutraceuticals exerted beneficial effects, possibly through their antioxidant actions, such as tocopherols, polyphenols, and phytoestrogens [112]. Despite progress made in recent years on nutraceutical therapy for hypertension and kidney disease, very few studies have targeted their potentials in reprogramming. Resveratrol is widely known as a natural phenolic compound with powerful antioxidant activity [113]. Resveratrol has been reported to increase NO bioavailability, down-regulate expression of NADPH oxidase, and enhance the antioxidant defense system [114,115]. There have been two reports demonstrating that resveratrol supplementation during pregnancy and lactation had BP benefits in adult offspring in a prenatal L-NAME exposure plus postnatal high-fat diet model [100] and a maternal bisphenol A plus high-fat diet model [101], both which are relevant for the reduction of oxidative stress damage. These studies support the notion that resveratrol supplementation might serve as a reprogramming strategy against the development of other adult diseases [116].

## 5. Conclusions and Future Perspectives

The interplay between maternal nutrition and oxidative stress is like a two-edged sword, leading to both harmful and beneficial effects in renal programming. This interplay in pregnancy on fetal programming is beginning to emerge in the literature [9,117]. Our review has highlighted suboptimal maternal nutrition not only affects oxidative stress, but it also has an impact on renal programming leading to adult kidney disease and hypertension. Early-life antioxidant nutrient intervention as a reprogramming strategy against the development of kidney disease and hypertension have been proven effective in animal models. Although these findings are promising, their clinical translation remains a critical challenge. Research in short-lived rodent models, with controlled interventions across their life span, provided key results revealing oxidative stress linking suboptimal maternal nutrition to renal programming underlying kidney disease and hypertension of developmental origins. In spite of several antioxidant nutrients showing beneficial effects, one major concern is their disparities in different animal models, species, doses, supplementation timing, and disease status. During the preparation of the current review, we found that various renal programming-related phenotypes and mechanisms of oxidative stress are not examined simultaneously in the same model. Also, the follow-up after the cessation of nutritional insults or antioxidant nutrient supplementations in most cited studies were just over a short period of time. Therefore, further research and more detailed clinical studies are needed to gain insight into the type of nutrients, the effective dosage, and the therapeutic period as the reprogramming therapy for kidney disease and hypertension of developmental origins.

## Figures and Tables

**Table 1:** Effects of early-life nutritional insults on renal programming, hypertension, and kidney disease in offspring in rodent animal models.

Animal Models	Intervention Period	Renal Phenotype	Low Nephron Number	Species/Gender	Age at Measure	Ref.
Caloric restriction						
30% caloric restriction	Pregnancy	↑BP	ND	Wistar/M + F	54 wk	[23]
50% caloric restriction	Pregnancy	↑BP	ND	Wistar/M + F	16 wk	[24]
50% caloric restriction	Pregnancy and lactation	↑BP, ↔GFR, glomerular hypertrophy, ↑tubulointerstitial injury	Yes	SD/M	12 wk	[25]
50% caloric restriction	Gestation day 10–21	↑BP	Yes	SD/M	26 wk	[26]
70% caloric restriction	Pregnancy	↑BP	ND	SD/M	16 wk	[27]
70% caloric restriction	Gestation day 0–18	↑BP	ND	Wistar/M + F	28 wk	[28]
Macronutrients						
20% w/v sucrose in drinking water	Pregnancy	↑BP	ND	SD/M	90 wk	[29]
High-fructose diet, 60%	Pregnancy and lactation	↑BP, ↔GFR	ND	SD/M	12 wk	[30]
High-fructose diet, 60%	Pregnancy and lactation	↑BP, altered renal transcriptome	ND	SD/M	12 wk	[31]
10% w/v fructose in drinking water	Pregnancy and lactation	↑BP	ND	C57BL6J mice/M	52 wk	[32]
Protein restriction, 6%	Pregnancy	↑BP	ND	SD/F	52 wk	[33]
Protein restriction, 8%	Lactation	↑BP,↑GFR, ↑Proteinuria	Yes	Wistar/M	150 day	[34]
Protein restriction, 8.5%	Pregnancy	↑BP	ND	SD/M	20 wk	[35]
Protein restriction, 8.5%	Pregnancy	↑BP, ↔GFR	Yes	SD/M	22 wk	[36]
Protein restriction, 8.5%	Pregnancy	↔BP, ↔GFR	No	SD/F	22 wk	[37]
Protein restriction, 9%	Pregnancy and lactation	↑BP	ND	Wistar/M	4 wk	[38]
Protein restriction, 9%	Pregnancy	↑BP	ND	Wistar/M	12 wk	[39]
Protein restriction, 9%	Pregnancy	↑BP	ND	Wistar/M + F	22 wk	[40]
Protein restriction, 9%	1 week before conception and throughout pregnancy	↑BP	ND	FVB/NJ mice/F	24 wk	[41]
High-fat diet, 24%	Lactation	↑BP	ND	Wistar/M	22 wk	[42]
High-fat diet, 25.7%	Lactation	↑BP	ND	SD/M + F	25 wk	[43]
High-fat diet, 45%	Pregnancy and lactation	↑BP	ND	C57BL6J mice/M	30 wk	[44]
High-fat diet, 58%	Pregnancy and lactation	↑BP	ND	SD/M	16 wk	[45]
High-fat diet, 58%	5 weeks before the delivery and throughout pregnancy and lactation	↔BP, ↓GFR, ↑glomerular injury, ↑tubulointerstitial injury, altered renal transcriptome	ND	SD/M	6 mo	[46]
Low-salt diet, 0.07%	Pregnancy and lactation	↑BP	ND	SD/M	21 wk	[47]
High-salt diet, 3%	Pregnancy and lactation	↑BP	ND	SD/M	21 wk	[47]
1% high-salt in drinking water	Pregnancy and lactation	↑BP, altered renal transcriptome	ND	SD/M	12 wk	[48]
Calcium-deficient diet	Pregnancy	↔BP, altered renal excretion	ND	WKY/M + F	52 wk	[49]
Magnesium-deficient diet	Pregnancy	↑BP	No	C57BL6J mice /M + F	24 wk	[50]
Mironutrients						
Zinc-deficient diet	Pregnancy and lactation	↑BP	ND	Wistar/M	12 wk	[51]
Iron restriction	4 weeks before conception and throughout pregnancy	↑BP	ND	RHL/M + F	10 wk	[52]
Iron restriction	4 weeks before conception and throughout pregnancy	↑BP	ND	Wistar/M + F	64 wk	[53]
Iron restriction	1 week before conception and throughout pregnancy	↑BP, glomerular hypertrophy, ↑tubulointerstitial injury	Yes	Wistar/M + F	18 mo	[54]
Vitamin D restricted diet	6 weeks before conception and throughout pregnancy and lactation	↑BP	ND	SD/M+F	8 wk	[55]
Imbalanced nutrients						
Methyl-deficient diet	Pregnancy and lactation	↑BP, altered renal transcriptome	ND	SD/M	12 wk	[56]
High methyl-donor diet	Pregnancy and lactation	↑BP, altered renal transcriptome	ND	SD/M	12 wk	[56]
High-fat diet, 45% plus 4% salt in drinking water	3 weeks before conception and throughout pregnancy and lactation	↑BP	ND	SD/M	19 wk	[57]
10% w/v fructose plus 4% salt in drinking water	4 weeks before conception and throughout pregnancy and lactation	↑BP	ND	SD/M	9 wk	[58]
High-fructose diet, 56.7% plus high-fat diet	Pregnancy and lactation	↑BP	ND	SD/M	16 wk	[59]
Multideficient diet	Pregnancy	↑BP, ↑GFR, glomerular hypertrophy	Yes	Wistar/M	100 day	[60]
Multideficient diet	Pregnancy	↑BP, ↓GFR	ND	Wistar/M	150 day	[61]
Early postnatal hypernutrition by reduction of litter size (3 pups/litter)	Lactation	↑BP, ↑GFR, glomerular hypertrophy	No	SD/M	22 mo	[62]

Studies tabulated according to nutritional intervention and age at measure. ND = Not determined; BP = blood pressure; GFR = glomerular filtration rate; Cr = creatinine; ↑ = increased; ↓ = decreased; ↔ = unaltered.; SD = Sprague−Dawley rat; M = Male; F = Female; RHL = Rowett Hooded Lister rat.

**Table 2:** Antioxidant nutrients used as reprogramming strategies to prevent hypertension and kidney disease of developmental origins.

Antioxidant Nutrients	Animal Models	Protective Effects	Species/Gender	Age at Measure (Week)	Ref.
Amino acids					
3% glycine in chow during pregnancy and lactation	Maternal protein restriction	Prevented hypertension	Wistar/M	4	[38]
2.5 g/L citrulline in drinking water in pregnancy and lactation	Maternal caloric restriction	Prevented reduced nephron number and renal dysfunction	SD/M	12	[25]
2.5 g/L citrulline in drinking water in pregnancy and lactation	Maternal L-NAME exposure	Prevented hypertension	SD/M	12	[89]
2.5 g/L citrulline in drinking water in pregnancy and lactation	Streptozotocin-induced diabetes	Prevented hypertension and kidney damage	SD/M	12	[80]
2.5 g/L citrulline in drinking water in pregnancy and lactation	Prenatal dexamethasone exposure	Prevented hypertension	SD/M	12	[90]
3% taurine in drinking water in pregnancy and lactation	Maternal high-sugar diet	Prevented hypertension and renal excretion function	SD rat/F	8	[91]
3% taurine in drinking water in pregnancy and lactation	Streptozotocin-induced diabetes	Prevented hypertension	Wistar/M and F	16	[92]
1% NAC in drinking water during pregnancy and lactation	Maternal L-NAME exposure	Prevented hypertension	SD rat/M	12	[79]
1% NAC in drinking water during pregnancy and lactation	Prenatal dexamethasone and postnatal high-fat diet	Prevented hypertension	SD rat/M	12	[85]
1% NAC in drinking water during pregnancy and lactation	Suramin-induced pre-eclampsia	Prevented hypertension	SD rat/M	12	[93]
NAC (500 mg/kg/day) in drinking water from gestational day 4 to postnatal day 10	Maternal nicotine exposure	Prevented hypertension	SD rat/M	32	[94]
BCAA-supplemented diets in pregnancy	Maternal caloric restriction	Prevented hypertension	SD/M	16	[27]
Fatty acids					
Conjugated linoleic acid in pregnancy and lactation	Maternal high-fat diet	Attenuated hypertension	SD/M	18	[95]
Vitamins and trace minerals					
Micronutrients (Vitamin C, E, selenium, and folic acid) by oral gavage in pregnancy	Maternal caloric restriction	Prevented hypertension	Wistar/M+F	16	[24]
Vitamin C (350 mg/kg/day) i.p. daily from gestational day 8 to 14	Prenatal LPS exposure	Prevented hypertension and proteinuria	SD/M	12	[96]
α-tocopherol (350 mg/kg body weight) daily during lactation	Multideficient diet	Prevented hypertension and renal dysfunction	Wistar/M	21	[61]
5 mg/kg folate in chow during pregnancy	Maternal protein restriction	Prevented hypertension	Wistar/M	15	[97]
Selenium (0.5 mg/kg) in chow from gestational day 14 to postnatal day 14	Maternal methimazole exposure	Prevented renal dysfunction	Wistar/M+F	2	[98]
Dietary fiber					
5% w/w long-chain inulin during pregnancy and lactation	Maternal high-fructose diet	Prevented hypertension	SD/M	12	[99]
5% w/w long-chain inulin during pregnancy and lactation	Perinatal high-fat diet	Prevented hypertension	SD/M	16	[45]
Nutraceuticals					
Resveratrol (50 mg/L) in drinking water during pregnancy and lactation	Prenatal L-NAME exposure plus postnatal high-fat diet	Attenuated hypertension	SD/M	16	[100]
Resveratrol (50 mg/L) in drinking water during pregnancy and lactation	Maternal exposure to bisphenol A plus high-fat diet	Prevented hypertension	SD/M	16	[101]

Studies tabulated according to types of antioxidant nutrients, animal models, and age at measure. BCAA = branched-chain amino acid. LPS = lipopolysaccharide. NAC = N−acetylcysteine. L−NAME = N^G^−nitro−L−arginine−methyl ester. SD = Sprague−Dawley rat. M = male. F = female.

## Abbreviations

ADMA	asymmetric dimethylarginine
BCAA	cranched chain amino acid
CAKUT	congenital anomalies of the kidney and urinary tract
CBS	cystathionine β-synthase
CSE	cystathionine γ-lyase
DDAH	dimethylarginine dimethylaminohydrolase
DEG	differential expressed gene
DOHaD	developmental origins of health and disease
GFR	glomerular filtration rate
GPx	glutathione peroxidase
H_2_S	hydrogen sulfide
L-NAME	N^G^-nitro-L-arginine-methyl ester
NAC	N-acetylcysteine
NGS	next-generation RNA sequencing
NO	nitric oxide
PRMT	protein arginine methyltransferase
RAS	renin-angiotensin system
RNS	reactive nitrogen species
ROS	reactive oxygen species
SDMA	symmetric dimethylarginine
SD	Sprague-Dawley rat
SOD	superoxide dismutase
3MST	3-mercaptopyruvate sulphurtransferase
